# Systematic Identification and Expression Analysis of the Sorghum *Pht1* Gene Family Reveals Several New Members Encoding High-Affinity Phosphate Transporters

**DOI:** 10.3390/ijms232213855

**Published:** 2022-11-10

**Authors:** Jinglong Zhang, Yixin Shen, Wei Chen, Binqiang Bai, Xiaomin Ji, Yingjun Chi

**Affiliations:** College of Agro-Grassland Science, Nanjing Agricultural University, Nanjing 210095, China

**Keywords:** Pht1, sorghum, high-affinity phosphate transporter, low-P condition

## Abstract

Sorghum (*Sorghum bicolor*) is known to have a more robust capability of phosphorus uptake than many other cereal plants, which could be attributed to its phosphate transporter 1 (Pht1) that has a high phosphorus affinity. There are eleven *SbPht1* genes in the sorghum genome, nine of which are expressed in sorghum roots or shoots in response to phosphorus deficiency (low-P). The molecular features of these nine genes were investigated by gene expression analysis, subcellular localization, and a yeast mutant complementation growth assay. They were found to be induced in response to low-P stress in root or shoot. All these SbPht1 proteins were found to be localized on the cell membrane, and SbPht1;8 was also detected in the endoplasmic reticulum. These SbPht1s were able to complement the yeast mutant EY917 that lacks all the functional phosphate transporters, and, among them, SbPht1;5, SbPht1;6 and SbPht1;8 could partially complement the yeast mutant strain EY917 in low-P conditions. Overall, these findings demonstrate that SbPht1;5, SbPht1;6, and SbPht1;8 are high-affinity phosphate transporters. SbPht1;5, in particular, is specifically involved in phosphorus uptake in the roots, whilst SbPht1;6 and SbPht1;8 are key players in both P uptake and P transport in response to low-P stress in sorghum.

## 1. Introduction

Phosphorus (P) is a macronutrient that plays manifold crucial roles in plant growth and development, as it is required for the synthesis of nucleotides, the cell membrane, and a vast number of phosphorylated primary and secondary metabolic compounds. Further, a sufficient availability of P favorably influences plant production, development rate, and resilience against various biotic and abiotic stresses [1]. The primary route by which plants obtain P is the uptake by the roots of soil inorganic phosphate (Pi). However, it is commonly considered that the available P in all soil types is generally insufficient for plant growth and the desired crop productivity [2]. Soils with a low total P content generally have poor retention of P fertilizers, whereas Pi is not always sufficient for plant utilization, even in soils with a high total P content, because a considerable amount of it is precipitated by interacting with aluminum and iron in acidic soils and with calcium in alkaline soils. The Pi concentration is typically greater than 10 mM in plant tissues, whereas it is less than 2 μM in soil [3,4,5]. Therefore, plants have evolved efficient transport systems for Pi absorption across the plasma membrane of root epidermal and cortical cells against a strong chemical potential gradient. Plant phosphate transporters (PTs), which are an important part of this system, have been shown to play a key role in P acquisition [1,6]. 

The recent classification of plant PTs based on their structures and subcellular compartmentation resulted in the designations of Pht1 through Pht5. Pht1 and Pht2 are usually found in the plasma membrane and chloroplasts, respectively, whilst the remaining three PTS, i.e., Pht3, Pht4, and Pht5, are typically located in the mitochondria, the Golgi apparatus, and the vacuole, respectively [7,8,9]. The plasma membrane protein Pht1 plays a pivotal role in Pi uptake by the plant roots from the soil. By virtue of their affinity for Pi, PTs have been dichotomized into high-affinity and low-affinity PTs [10]. The high-affinity PTs may translocate Pi from a P-limited external medium to the cytoplasm [11], suggestive of their effectiveness in P acquisition under low-P stress [12]. Many of the PTs with the highest P affinity are members of the Pht1 family [7,13]. Pht1 is, therefore, regarded as the most important carrier for P from the soil in low-P conditions. Consequently, considerable focus has been placed on Pht1 family genes in recent years, which has resulted in their identification and functional analysis in an eclectic array of plant species, such as *Arabidopsis thaliana* [14,15,16], *Solanum lycopersicum* [17,18,19], *Medicago truncatula* [20,21], *Solanum tuberosum* [22,23], *Oryza sativa* [24,25], *Zea mays* [26], *Triticum aestivum* [27,28], *Setaria italica* [29], and Glycine max [30]. 

Not only is sorghum the staple food for more than 500 million people, but it is also a popular silage crop [31,32,33]. Prior research determined that sorghum’s capacity for Pi uptake is significantly greater than that of maize [34]. Intriguingly, eleven *Pht1* genes have been identified in the sorghum reference genome, whereas 13 *Pht1* genes have been identified in maize, implying that biochemical properties may play a primary role in determining their function. This is well in line with numerous previous studies, which demonstrated that the post-transcriptional regulation of PTs and their biochemical properties influence their functional activity more than the sheer number of genes and gene expression levels, as demonstrated in flax, which was shown to uptake more P than other plant species from the common mycorrhizal network in soil [35]. This necessitates the identification and characterization of the *Pht1* gene family in individual plant species, sorghum in this case, as opposed to presuming they all behave the same. 

In this study, we cloned several *Pht1* genes that are expressed in sorghum root under low-P conditions. The characteristics of these genes were investigated by gene expression analysis, subcellular localization, and functional complementation via ectopic expression in a yeast mutant that lacks PT activity. Our results demonstrate that *SbPht1;5*, *SbPht1;6,* and *SbPht1;8* encode high-affinity PTs in sorghum. SbPht1;5, in particular, is specifically involved in P uptake in the root, whilst SbPht1;6 and SbPht1;8 play a more general role in P uptake and P transport in sorghum in response to low-P stress. Furthermore, SbPht1;8 was discovered in the endoplasmic reticulum as well as in the plasm membrane, implying that it plays a role distinct from that of other SbPht1s. Overall, these findings improve our understanding of P uptake, trafficking, and regulation, shed additional light on the P transport mechanism, and therefore may be conducive to bolstering P utilization efficiency in sorghum.

## 2. Results

### 2.1. Identification of Putative SbPht1 Genes

Eleven putative *Pht1* genes, designated *SbPht1;1* through *SbPht1;11*, were identified from the sorghum reference genome (inbred line BT×632) (Table 1). They encode proteins varying from 510 to 554 amino acids, with the predicted molecular weights varying from 56.25 kDa to 60.42 kDa, and the predicted isoelectric point varying from 6.67 to 9.52.

Gene-specific primers were designed to isolate the putative *SbPht1s* by PCR. A total of nine target fragments were obtained from the sorghum roots upon low-P treatment, the sizes of which were consistent with the putative *SbPht1s*. Following the nomenclature in rice, they were identified as *SbPht1;1*, *SbPht1;2*, *SbPht1;3*, *SbPht1;4*, *SbPht1;5*, *SbPht1;6*, *SbPht1;8*, *SbPht1;9* and *SbPht1;11* after DNA sequencing and sequence alignment (Figure S1). *SbPht1;7* and *SbPht1;10* could not be isolated from the roots and shoots under this experimental condition.

### 2.2. Bioinformatics Analysis of Pht1 Transporters from Sorghum

**Figure 1:**
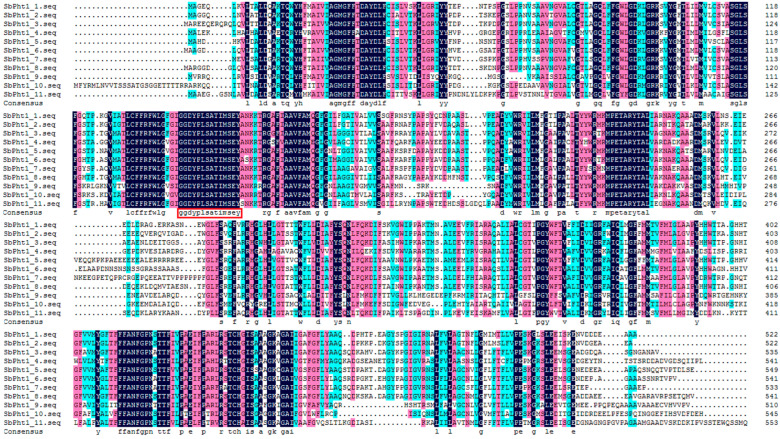
Multiple alignment of the amino acid sequences of SbPht1s.

**Figure 2:**
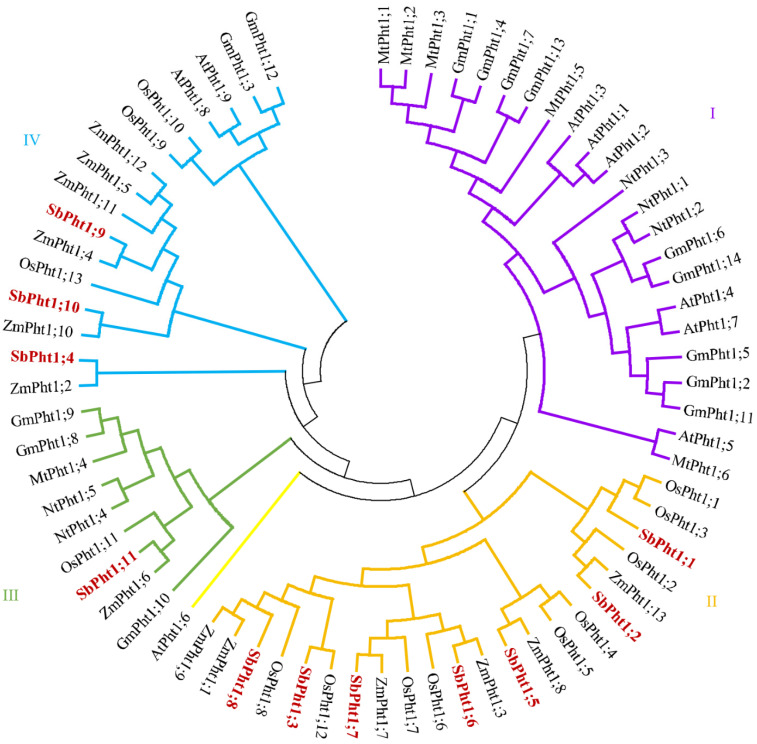
Phylogenetic analysis of Pht1. The phylogenetic analysis was based on the putative amino acid sequences of PTs deduced from their DNA sequences. The sequences retrieved from the NCBI GenBank are listed in Table S1.

Amino acid sequence analysis revealed that SbPht1s are endowed with a conserved domain, GGDYPLSATIMSE (Figure 1), and 9–12 transmembrane helices (Figure S2). A phylogenetic tree of Pht1 (GenBank accession numbers are listed in Table S1) was constructed by the neighbor-joining method, which siloed the Pht1s into five distinct groups (Figure 2). SbPht1;1, SbPht1;2, SbPht1;3, SbPht1;5, SbPht1;6, SbPht1;7, SbPht1;8 were assigned to Class II (Figure 2), which includes only monocotyledonous Pht1s, most of which were not induced by arbuscular mycorrhizal (AM) [26,36]. SbPht1;11 was classified in Class III, which contains Pht1s from both monocotyledonous and dicotyledonous species, which were induced by AM [26,36]. SbPht1;4, SbPht1;9, and SbPht1;10 were classified into Class IV, which contains Pht1s from both monocotyledonous and dicotyledonous species, which were partially induced by AM [26,37].

### 2.3. Expression Analysis of SbPht1s in Responss to Low-P Stress

**Figure 3:**
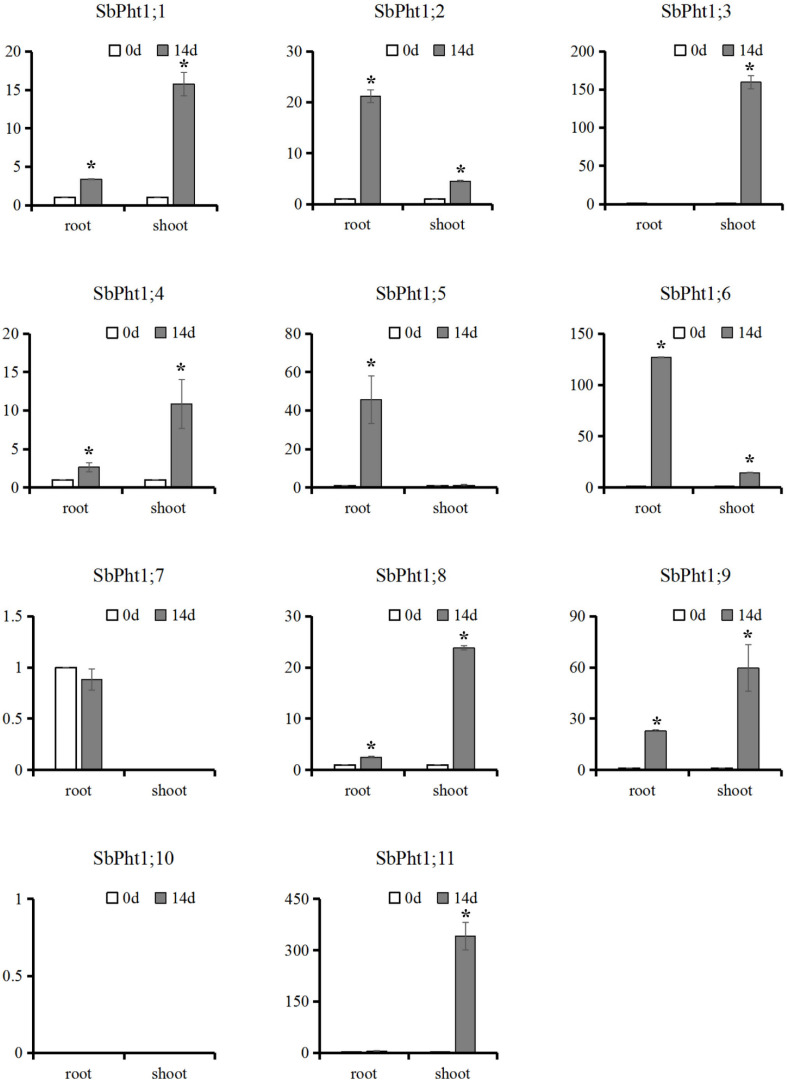
Expression of SbPht1 in root and shoot of sorghum upon low-P stress treatment for 14 days (d). * represents Tukey’s test significance at the threshold of *p* < 0.01.

The expression of *SbPht1s* was profiled using qRT-PCR upon low-P treatment for 14 d in hydroponic culture. As is evident in Figure 3, *SbPht1;5* was significantly induced by the low-P condition only in roots (*p* < 0.01), whereas *SbPht1;3* and *SbPht1;11* were significantly induced by low-P stress only in shoots (*p* < 0.01), and the remaining five *SbPht1* genes were significantly induced by low-P stress in both root and shoot tissues (*p* < 0.01).

### 2.4. Subcellular Localization of SbPht1s

**Figure 4:**
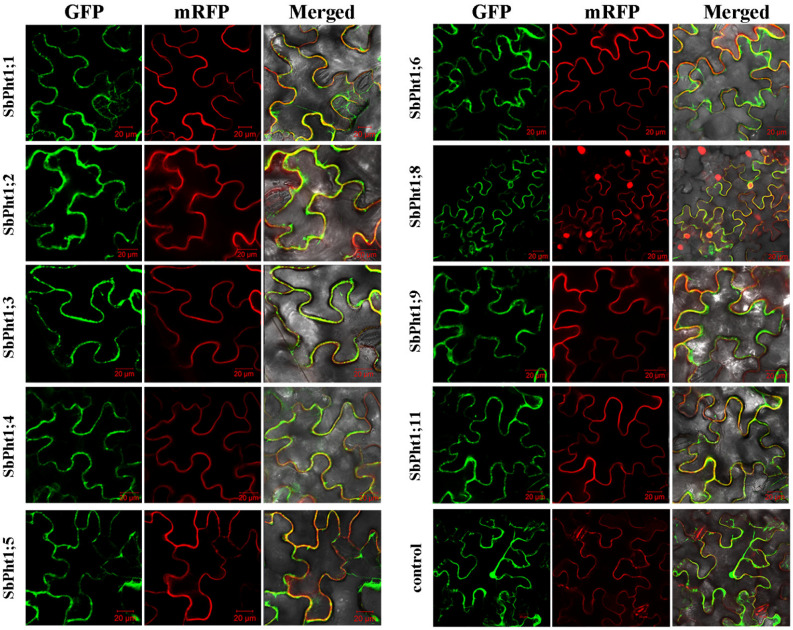
Subcellular localization of SbPht1s. SbPht1;1-GFP, SbPht1;2-GFP, SbPht1;3-GFP, SbPht1;4-GFP, SbPht1;5-GFP, SbPht1;6-GFP, SbPht1;8-GFP, SbPht1;9-GFP, and SbPht1;11-GFP. The fluorescence signals overlapped with the signal of the mRFP cell membrane marker.

The subcellular location of the nine isolated *SbPht1s* was assayed using a transient expression system in *N. benthamiana* leaves. The open reading frame of each *SbPht1* was cloned into the vector pFGC5941–GFP by in-frame fusion with *GFP* and generated nine SbPht1s-GFP reporter genes. Along with the empty vector, each of them was co-infiltrated with the PIP2α plasma membrane marker into the leaves of *N. benthamiana*. At 2 d post infiltration, under a confocal laser microscope, the GFP signal was observed to overtly overlap with the mRFP plasma membrane marker, whereas the GFP signal in the leaves infiltrated with pFGC5941–GFP was dispersed across the entire cell (Figure 4). These data showed that the plasma membrane is the subcellular localization site of all nine SbPht1s (Figure 4).

**Figure 5:**
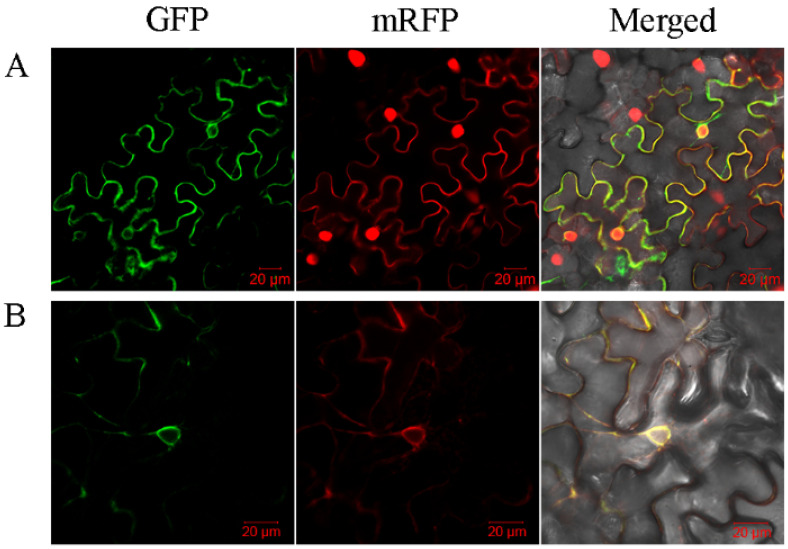
Subcellular localization of SbPht1;8. (**A**) SbPht1;8-GFP fluorescence signal overlapped with those of the mRFP nucleus and cell membrane markers; (**B**) SbPht1;8-GFP fluorescence signals overlapped with those of the mRFP endoplasmic reticulum and cell membrane markers.

The GFP signal in SbPht1;8-GFP-infiltrated leaves was also discernible around the nucleus. For clarification, a further leaf infiltration experiment was conducted using nuclear and endoplasmic reticulum markers. It was shown that the fluorescent signal around the nucleus coincided with the fluorescent signal of the endoplasmic reticulum marker but not with that of the nuclear marker (Figure 5), demonstrating that SbPht1;8 is also an endoplasmic reticulum-localized protein.

### 2.5. Functional Analysis of SbPht1s in Yeast

**Figure 6:**
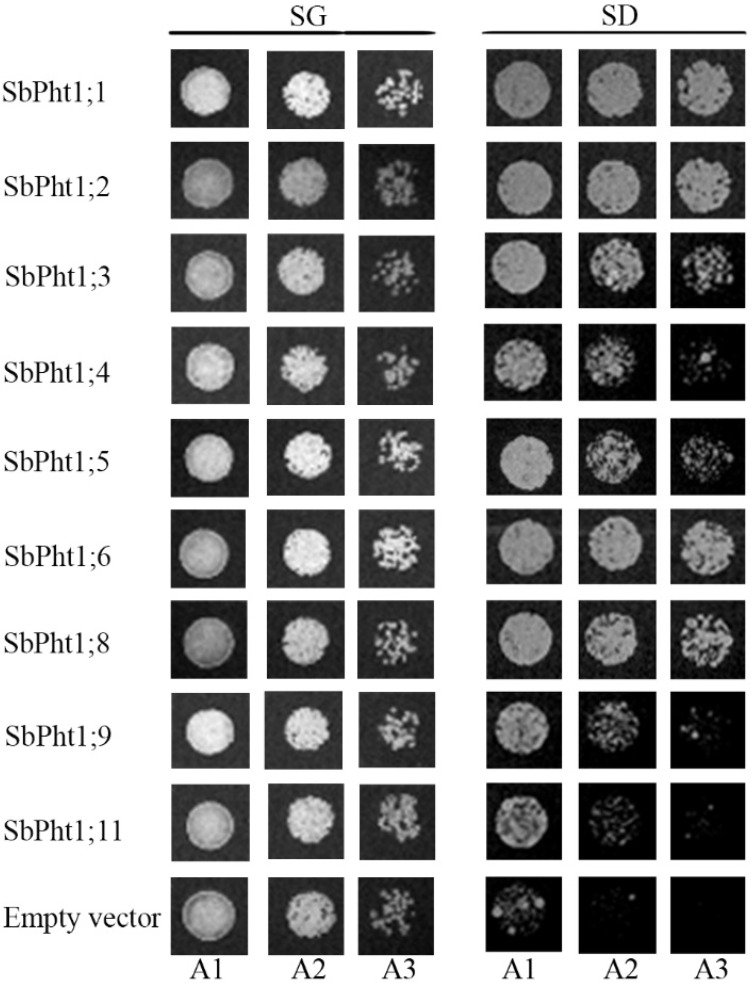
Functional expression of nine *SbPht1* genes in yeast in the presence of sufficient P. The concentrations of A1, A2, and A3 were diluted 10, 100, 1000 times, respectively.

The nine *SbPht1s* cloned from sorghum seedings were functionally analyzed using the yeast mutant EY917, which lacks all the five functional PTs (Δpho84/Δpho87/Δpho89/Δpho90/Δpho91) and is therefore unable to transport Pi [36]. EY917 contains a plasmid harboring a *Pho84* gene driven by the GAL1 promoter, enabling its normal growth on a medium with galactose as the sole carbon resource. Positively transformed yeast strains were cultured on the synthetic media SD or SG in the presence of sufficient P. It was shown that EY917 cells ectopically expressing any of the nine *SbPht1s* were able to grow normally on SG, akin to those expressing the empty vector. However, on the SD medium, their growth appeared to be conspicuously more robust than that of the empty-vector control (Figure 6). It is therefore evident that all the nine SbPht1s isolated from sorghum roots could complement the yeast PTs function in the presence of sufficient P.

**Figure 7:**
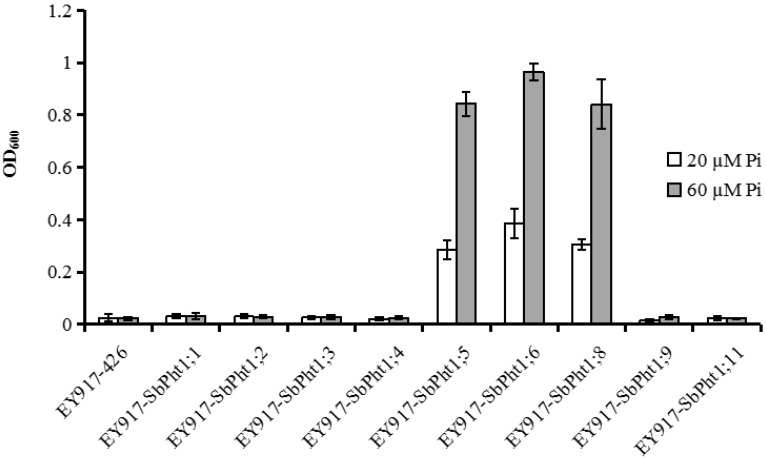
Functional complementation of nine *SbPht1* genes by ectopic expression in yeast mutant strain EY917 under P-deficient conditions.

However, under two different low-P (20 and 60 µM) conditions, the yeast cells ectopically expressing *SbPht1;5*, *SbPht1;6* and *SbPht1;8* grew and thrived better than those expressing the empty vector control, whereas those overexpressing other *SbPht1s* resembled the control (Figure 7). This showed that SbPht1;5, SbPht1;6 and SbPht1;8 could complement the yeast PTs under low-P conditions and thus manifested a higher P affinity than other PTs in sorghum.

## 3. Discussion

In plants, PTs are essential for P uptake and P transport. As membrane-bound proteins, the Pht1 family members are commonly located on the cytoplasmic membrane, acting as the primary vehicle in plants to acquire P from the soil. The number of PTs on the cytoplasmic membrane and their affinity for P have a direct effect on the efficiency of P uptake [38]. Identifying the SbPht1 members that are endowed with a high affinity for P is crucial for elucidating the process of P acquisition and transport in sorghum. 

### 3.1. Identification of SbPht1 Family Genes

There are five families of PTs: Pht1, Pht2, Pht3, Pht4, and Pht5, among which Pht1 is localized on the cytoplasmic membrane and plays a crucial role in plant P uptake from the soil [39]. Like rice and maize, sorghum is a graminaceous plant species. Eleven *Pht1s* genes were identified in sorghum, while thirteen *Pht1s* genes were identified, respectively, in rice and maize. However, P uptake by sorghum was much higher than that by maize [34]. Moreover, the sequence of *SbPht1;10* shares high homology with that of its ortholog, *ZmPht1;10*, but their expression patterns are very different [26]. The association between individual Pht1s and their P affinity may vary from species to species and invokes for the study of individual crops before using them as important gene tools for crop improvement.

In this study, we identified nine *SbPht1s* that are expressed in sorghum roots and which display a high degree of sequence identity with their paralogs in sorghum and with orthologs in other plant species. The deduced protein sequences are endowed with conserved hydrophobic domains and multiple transmembrane helices that are characteristic of the known plant Pht1s [13,18,24,40]. The fact that all nine *SbPht1s* were found in the plasma membrane or in the endoplasmic reticulum is consistent with their membrane-bound nature. The yeast complementation experiments illustrated that all the nine *SbPht1s* could complement the yeast mutants deficient in P uptake, demonstrating their functional role in this process.

### 3.2. SbPht1;5, SbPht1;6, and SbPht1;8 Play Important Roles in Sorghum in Response to Low-P Stress

Studies on Pht1 in different plant species revealed that the *Pht1* genes that are primarily induced in the roots by low-P stress are responsible for P absorption, whilst those expressed in both roots and shoots are involved in P transport and redistribution, in addition to P uptake [14,41,42,43,44]. The root-specific upregulation of *SbPht1;5* and the shoot-specific upregulation of *SbPht1;3* and *SbPht1;11* are indicative of these genes’ potential specific roles in P acquisition from the soil and in P transport in the shoot, respectively. In contrast, the expression of the other six *SbPht1* members, including *SbPht1;1*, *SbPht1;2*, *SbPht1;4*, *SbPht1;6*, *SbPht1;8*, and *SbPht1;9*, was induced in both the root and the shoot in response to low-P stress, suggesting that they participate in both P uptake from the soil and P transport across different plant tissues.

It is a common practice to study the functional role of plant PTs in the yeast mutant EY917. It was shown that all nine root-expressed SbPht1s were able to partially complement the yeast P uptake mutant in a sufficient-P environment, but only SbPht1;5, SbPht1;6, and SbPht1;8 were able to do so in a low-P environment, suggesting that these three genes encode high-affinity PTs in sorghum. We also demonstrated that SbPht1;5 plays a primary role in P uptake in roots, whilst SbPht1;6 and SbPht1;8 can improve P uptake and P transport under low-P conditions in sorghum. These findings are well in line with two previous studies on rice, where OsPht1;6 and OsPht1;8, the respective orthologs of SbPht1;6 and SbPht1;8, were identified as high-affinity PTs that are induced by low P levels in both roots and shoots and are involved in both P uptake and P transport throughout the entire growth period [25,45]. In rice, OsPht1.8 was strongly expressed in roots, stems, leaves, endosperm, seed shells, and anthers and was involved in the regulation of root architecture in response to low-P stress, in addition to its effect on grain filling by regulating the P transport [45,46]. In our study, it is intriguing to note that SbPht1;8 was the only SbPht1 member that was localized in the endoplasmic reticulum in addition to the plasma membrane. The functional specificity of SbPht1;8 in P homeostasis in plants is intriguing but elusive and warrants further investigation. 

### 3.3. SbPht1;7 and SbPht1;10 May Have Divergent Functions

The absence of SbPht1;7 and SbPht1;10 in the shoots and roots of sorghum under the specific experimental conditions of this study implies for them a specific role that is distinct from that of other SbPht1s expressed in roots and shoots. A previous study showed that *SbPht1;10* was induced by AM, suggesting that it may act in concert with AM to acquire and/or distribute Pi [35]. It is therefore plausible that *SbPht1;10* is only expressed in response to AM induction. The fact that it was not induced by the low-P treatment is intriguing but elusive and remains to be investigated. In Arabidopsis, AtPht1;6 was undetectable in the roots and shoots and was not inducible by low-P stress; rather, it was found to play a role in supplying P to flowers [14,41], inkling an area that could be investigated for the potentially divergent role of SbPht1;7. Future studies of different SbPht1 family members for a more nuanced understanding of their diversified and versatile functionalities are clearly warranted.

## 4. Materials and Methods

### 4.1. Plant Materials and Growth Condition

The sorghum (*Sorghum bicolor*) accession 12,484 was used for *SbPht1* isolation. The seeds of sorghum were surface-sterilized with 75% ethanol for 5 min, blotted dry, and then germinated on moist filter paper prior to being transferred to distilled water for plant establishment. At the three-leaf stage, the seedlings were transferred to either a sufficient-P condition (Hoagland solution with 1.0 mmol/L KH_2_PO_4_) or a low-P condition (Hoagland solution with 1 μmol/L KH_2_PO_4_ and 1.0 mmol/L KCl) and cultured for two weeks in a growth chamber with a 12 h light (28 °C)/12 h dark (22 °C) cycle. The nutrient solution (pH = 5.8) was renewed every three days (d). There were three replicates of each treatment. Every two days, roots and shoots exposed to different P concentrations were harvested and frozen at −80 °C for the subsequent experiments.

### 4.2. Isolation of Pht1s from Sorghum

The *S. bicolor* genome database (http://plantgdb.org/ accessed on 15 March 2020) was blasted with the previously identified plant *PT* genes, including those from *A. thaliana*, *Oryza sativa,* and *S. bicolor*. After removing overlapping sequences, the remaining genes were verified in the Pfam database (http://pfam.xfam.org/ accessed on 25 March 2020). At 2, 4, 6, 8, 10, 12, and 14 d, the root and shoot tissues of *S. bicolor* accession 12,484 grown under sufficient-P or low-P conditions were collected. The total RNAs were extracted using a Plant RNA Extraction Kit (TIANGEN, Beijing, China). The first strand of cDNA was synthesized by using the HiScript II 1st Strand cDNA Synthesis Kit (Vazyme, Nanjing, China). Specific primers were designed and synthetized based on the putative *Pht1* gene sequences in *S. bicolor* (Table S2). All the attained *SbPht1* genes were verified using DNA sequencing.

### 4.3. Bioinformatics Analyses

The transmembrane (TM) segments were predicted using the program TMHMM (http://www.cbs.dtu.dk/services/TMHMM/ accessed on 25 March 2020). The deduced SbPht1 sequences were aligned, together with their homologs from other plant species, by the ClustalW with default parameters, and a phylogenetic tree was constructed by MEGA 5 using the neighbor-joining method after a preliminary assessment of the methodologies (data not presented). 

### 4.4. Gene Expression Analysis

Quantitative reverse transcriptase–polymerase chain reaction (qRT-PCR) was performed by the AceQ^®^ qPCR SYBR^®^ Green Master Mix (Vazyme). The PCR was conducted with a pre-denaturation at 95 °C for 5 min, followed by 40 cycles at 95 °C for 10 s and 60 °C for 30 s. Based on the evaluation of two different validation programs, including GeNorm and NormFinder, *Sb18S rRNA* was selected as an internal control gene, which was identified as the most stably expressed gene among five tested reference genes (*18S rRNA*, *Action*, *EIF4a*, *GAPDH*, *UBQ10*), as shown in Figure S3 [47,48]. The relative expression value of the *SbPht1* genes at a specific time under low-P treatment is presented as fold changes compared to the untreated sample at the same time using the 2^−∆∆Ct^ method [49]. There were three independent biological replicates. All the primers used for qRT-PCR are listed in Table S3.

### 4.5. Subcellular Location

For the subcellular localization assay, the entire coding region without the stop codon of *SbPht1s* was amplified using specific primers (Table S4), and the resultant PCR product was cloned into the vector pFGC5941–GFP to generate the recombinant construct SbPht1:GFP using the recombinase-dependent single-fragment cloning kit ClonExpress^®^ II One Step Cloning Kit (Vazyme). Following DNA sequencing confirmation, the resulting vectors were transformed into *Agrobacterium tumefaciens* strain GV3101, which was used to infiltrate *Nicotiana benthamiana* leaves as previously described [50]. Two days after infiltration, the GFP fluorescence signals in the infiltrated leaves were imaged using a confocal laser scanning microscope LSM510 (Carl Zeiss, Oberkochen, Germany) [51]. The plasma membrane-specific AtPIP2A was used as a control [52].

### 4.6. Yeast Mutant Complement Growth Assay

The yeast (*Saccharomyces cerevisiae*) mutant strain EY917 that is defective in all the five functional *PTs* (Δpho84/Δpho87/Δpho89/Δpho90/Δpho91) was used for the functional complementation assay [36]. EY917 contains a plasmid harboring a *Pho84* gene under the transcriptional control of the GAL1 promoter, permitting the normal growth of this strain on synthetic media using galactose as the sole carbon resource [36]. 

Then, the open reading frames of the *SbPht1;1, SbPht1;2, SbPht1;3, SbPht1;4, SbPht1;5, SbPht1;6, SbPht1;8, SbPht1;9 and SbPht1;11* genes were amplified from the cDNAs derived from the seedlings of sorghum accession 12484 and were then cloned into the pENTR/D-TOPO vector (Invitrogen, Carlbad, CA, USA) using specific primers (Table S5). The clones harboring each of these vectors were selected by a PCR assay, and the vectors’ authenticity and integrity were further verified by DNA sequencing. Each of these vectors was then recombined with the destination vector, pAG426GPD-ccdB, using the Gateway system as previously described [53]. As the vector pAG426GPD-ccdB contains *Ura3* driven by a constitutive promoter, EY917 cells transformed with the recombinant constructs, pAG426GPD-*SbPht1s,* were selected on a synthetic medium supplemented with galactose (SG) without Ura, while the empty vector pAG426GPD was used as a negative control. 

The transformed yeast cells were harvested by centrifugation, washed with 3% glucose once and with sterile water for three times prior to adjusting their concentration to an OD_600_ of 0.5. A 3 µL aliquot of the serial dilutions of 10, 100, 1000 times was spotted onto the SG or the synthetic medium-supplemented glucose (SD) agar plates and incubated at 30 °C for 3 d. The cells were also transferred to SD liquid medium containing different Pi concentrations (20 and 60 µM) and incubated at 30 °C for one week, which was followed by cell density measurements at OD_600_.

## Figures and Tables

**Table 1:** Pht1 gene family in sorghum.

Gene	Alias	Accession Number	CDS Size (bp)	Amino acid Length (AA)	Protein Molecular Mass (Da)	Isoelectric Point of Protein
*SbPht1;1*	*Sb01g020580*	XM_002467113.2	1566	522	56,952.80	7.85
*SbPht1;2*	*Sb01g046890*	XM_002465800.2	1566	522	57,129.30	8.69
*SbPht1;3*	*Sb01g046900*	XM_002468450.2	1605	535	57,871.40	8.74
*SbPht1;4*	*Sb02g009880*	XM_002461845.1	1623	541	58,903.60	7.95
*SbPht1;5*	*Sb06g002800*	XM_002447480.2	1647	549	60,197.30	7.04
*SbPht1;6*	*Sb07g023780*	XM_002445623.2	1623	541	57,785.30	8.88
*SbPht1;7*	*Sb01g047910*	DQ459071.1	1599	533	57,492.10	6.92
*SbPht1;8*	*Sb01g020570*	XM_002464513.2	1623	541	58,768.40	7.82
*SbPht1;9*	*Sb06g002560*	XM_002446106.2	1530	510	56,245.90	9.52
*SbPht1;10*	*Sb06g002540*	XM_002447472.2	1635	545	60,421.60	6.67
*SbPht1;11*	*Sb03g029970*	XM_002458208.2	1662	554	60,245.10	7.94

## Data Availability

Not applicable.

## Supplementary Materials

The following supporting information can be downloaded at: https://www.mdpi.com/article/10.3390/ijms232213855/s1, Figure S1: Amplification of SbPht1. 1–11 represent SbPht1;1-SbPht1;11 in turn. M represents DL 2000 DNA maker; Figure S2: Prediction of SbPht1 transmembrane domains; Figure S3: The stability of five reference genes in different S. bicolor organs; Table S1: GenBank of sequences for phylogenetic analysis; Table S2: Gene-specific primers used for isolation of Pht1s in Sorghum; Table S3: Gene-specific primers used for gene expression analysis by qPCR; Table S4: Gene-specific primers used for construction of fusion expression vector for subcellular localization; Table S5: Gene-specific primers were used to clone *SbPht1* genes into pENTR/D-TOPO vector for yeast mutant complement growth assay.
