# Supplementary figures and images for: Systematic Identification and Expression Analysis of the Sorghum Pht1 Gene Family Reveals Several New Members Encoding High-Affinity Phosphate Transporters
Source: Int J Mol Sci. 2022 Nov 10;23(22):13855. doi: 10.3390/ijms232213855 (PMC9698377; doi:10.3390/ijms232213855)

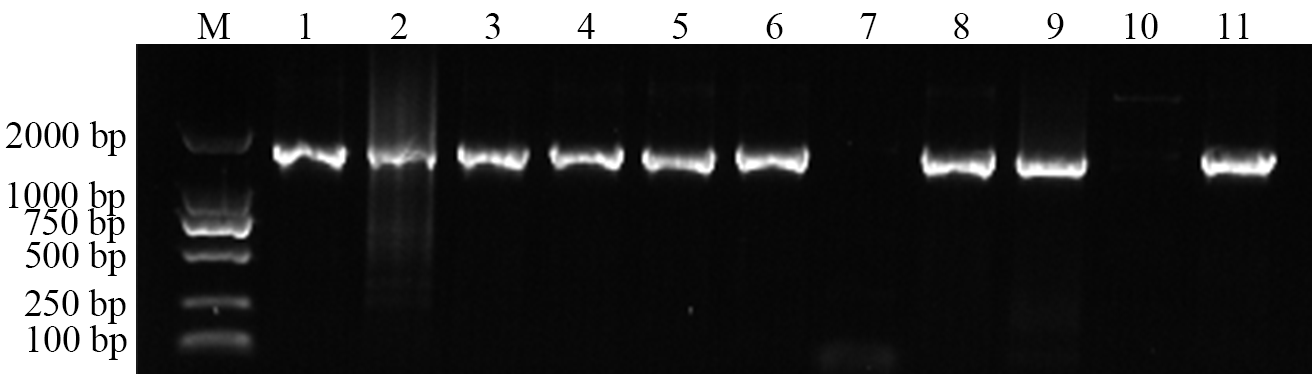

Supplement: Supplementary file 1 [file ijms-23-13855-s001.zip › Figure S1.tif]

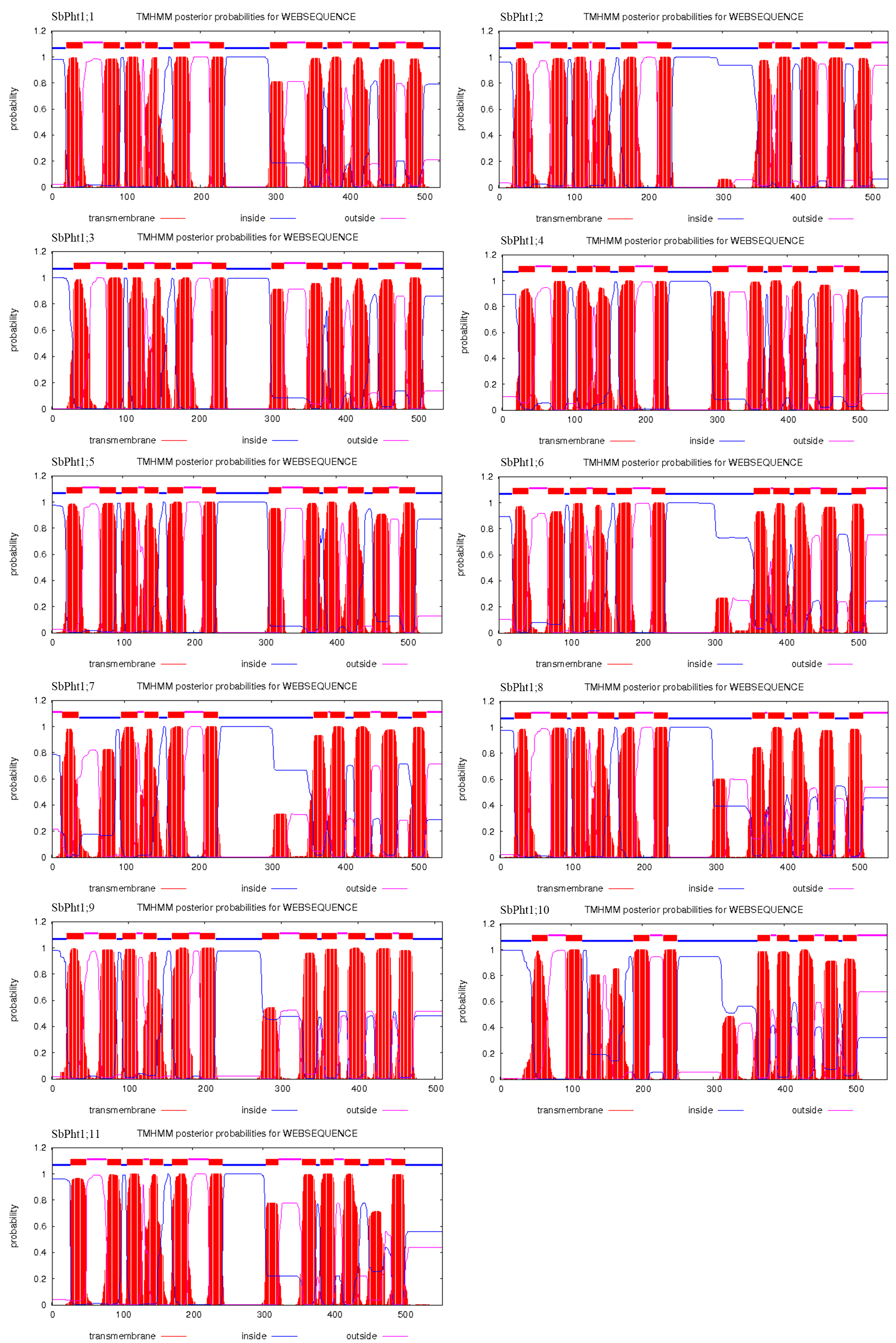

Supplement: Supplementary file 1 [file ijms-23-13855-s001.zip › Figure S2.tif]

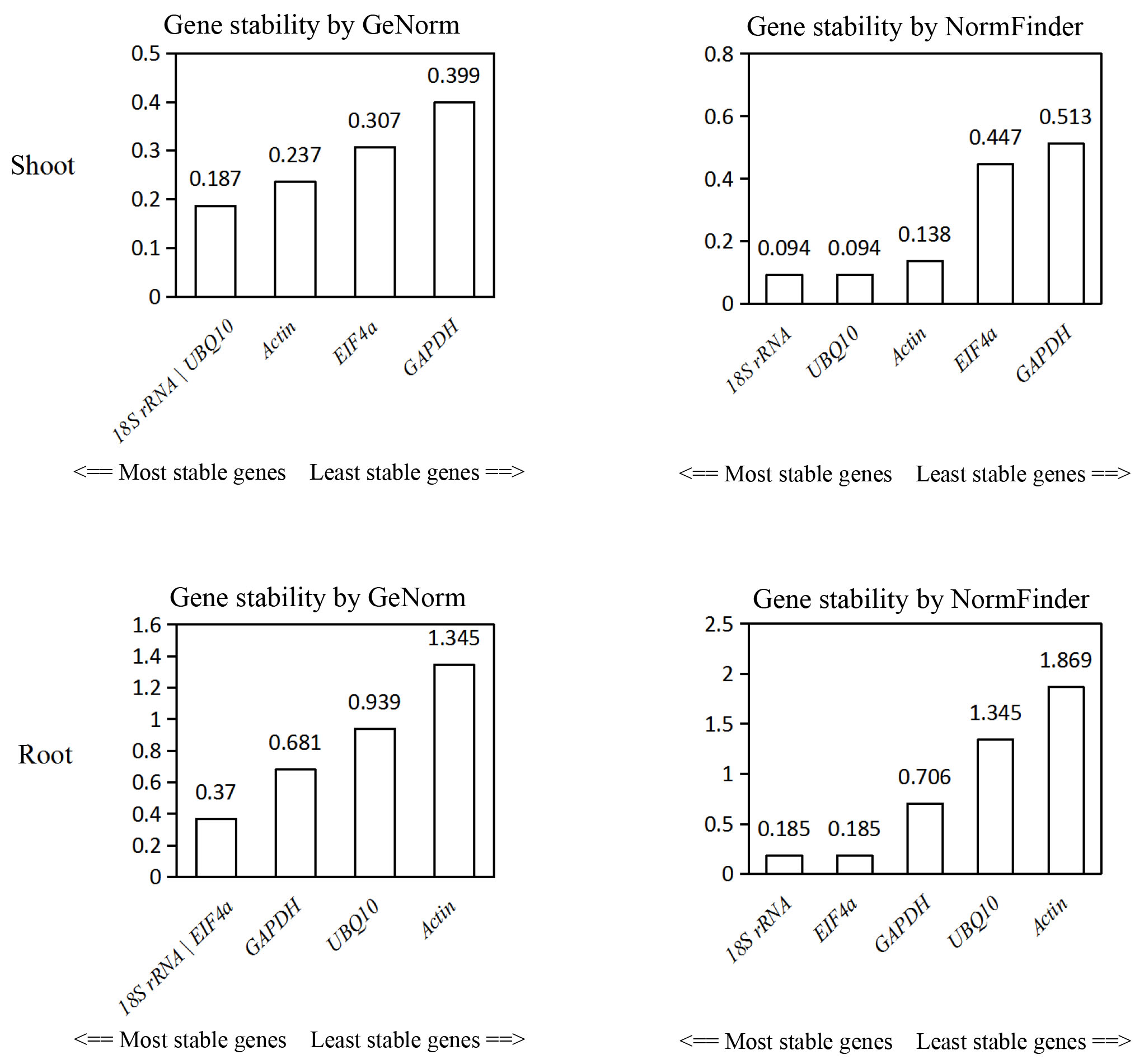

Supplement: Supplementary file 1 [file ijms-23-13855-s001.zip › Figure S3.jpg]
